# Exercise in the Prevention and Management of Oxidative Stress-Linked Diseases

**DOI:** 10.1155/2018/7643721

**Published:** 2018-04-26

**Authors:** Paola Venditti, Yong Zhang, Zsolt Radák, José Magalhães

**Affiliations:** ^1^Department of Biology, University Federico II of Naples, Naples, Italy; ^2^Tianjin Key Laboratory of Exercise Physiology and Sports Medicine, Tianjin University of Sport, Tianjin, China; ^3^Faculty of Physical Education and Sport Science Research Institute of Sport Science, Semmelweis University, Budapest, Hungary; ^4^Department of Sport Biology Faculty of Sport, University of Porto, Porto, Portugal

It is well established that health preservation and prevention of age-related disorders require the adoption of appropriate lifestyles including a habitual exercise regimen. Regular aerobic physical activity (training) protects against stress conditions inducing, in almost all body tissues, metabolic and biochemical adaptive responses. Among such responses there is the increased capacity to counteract conditions in which the production of reactive oxygen species (ROS) increases, including prolonged or strenuous exercise. It is now established that several physiopathological conditions, including aging, are associated with the increase in ROS-induced cellular damage. Moreover, current aerobic physical activity is recommended as an adjunctive therapy to mitigate the adverse effects of some pathologies.

In the present special issue, papers are reported exploring the effects on humans and animals of exercise training as a mean to slowing down the degenerative processes occurring with aging or to mitigate the effects of neurodegenerative or cardiovascular diseases.

Thus, Daniele et al. studied the effects of aging and physical exercise on the epigenetic modifications of the *α*-synuclein gene (*SNCA*) in health subjects. They explored whether age and physical activity are related to blood intron1-*SNCA* (*SNCAI1*) methylation, as well as further parameters linked to such epigenetic modification (total, oligomeric *α*-synuclein, and DNA methyl transferase concentrations in blood). The methylation degrees were correlated with the amount of total and oligomeric *α*-synuclein content in red blood cells, selected as a valid cellular model because they accumulate *α*-synuclein and are particularly susceptible to oxidative stress. They found that the *SNCAI1* methylation status increased with aging, and consistent with this result, low *α*-synuclein levels were found in blood. In this population, higher physical activity reduced the total and oligomeric *α*-synuclein levels.

Pilch et al. studied the effects of a relatively new and popular activity, the Nordic Walking (NW), on somatic indices, oxidant and antioxidant status, and interleukin and calcidiol levels in middle-aged women after a 12-week NW training program. They found that the NW training led to a significant decrease of the total body mass and fat mass and to an increase in lean body mass. It also contributed to a significant increase in total antioxidative status and calcidiol levels. Reverse correlations between IL-6 and total oxidative capacity levels and between IL-6 and calcidiol levels were found before and after training, respectively. They concluded that NW training undertaken by premenopausal women not only has a positive effect on body composition but also on the plasma antioxidative capacity.

Other papers deal with the effects of aerobic training on animal models of illness.

F. A. Guarnier et al. evaluated the effects of 2 months treadmill running on an animal model of Calsequestrin-1 knockout (CASQ1-null) mice, showing an elevated sensitivity to strenuous exercise and environmental heat, characterized by crises known as exertional/environmental heat strokes. Training reduced the mice mortality due to 1 hr. heat exposure (41°C). The protective training effect was accompanied by reduced mitochondrial damage and improvement of their function and lowering in both calpain activity and lipid peroxidation in membranes isolated from sarcoplasmic reticulum and mitochondria. The authors suggest that the protective effect of aerobic training is mediated by a reduction in oxidative stress during exposure of CASQ1-null mice to adverse environmental conditions.

D. J. Flis et al. used transgenic mice with the G93A hmSOD1 gene mutation as model of amyotrophic lateral sclerosis (ALS) to study the effects of swim training on the progression of ASL. They evaluated the training effects on the cell structures formed by the mitochondria and endoplasmic reticulum membranes (MAMs). They evaluated muscle energy metabolism, oxidative stress parameters, Caveolin-1 protein levels, and cholesterol content in crude mitochondrial fraction in mice with different disease progression and training status. The progression of ALS was related to the lowering of Caveolin-1 protein levels and cholesterol accumulation in a crude mitochondrial fraction. These changes were associated with aerobic and anaerobic energy metabolism dysfunction and higher oxidative stress. The data indicate that swim training prolongs the lifespan of ALS mice reducing the changes in MAMs components. Swim training also maintained mitochondrial function and lowered oxidative stress. Their data suggest that modification of MAMs might play a crucial role in the exercise-induced deceleration of ALS development.

Lambert et al. used rats with high fat diet-induced obesity and insulin resistance to study the effects on different white adipose tissue depots of (i) nutritional supplementation with grape polyphenols (50 mg/kg/day), (ii) treadmill exercise training (1 hr./day, 5 days/wk.) for 8 weeks, and (iii) a combination of exercise and grape polyphenol supplementation. They showed that both polyphenol supplementation and exercise decreased the amount of adipose tissue depots, and mesenteric inflammation. Moreover exercise showed to be more effective in counteracting insulin resistance, but their combination did not show cumulative benefit.

Belaya et al. examined the effects of aging and long-term wheel running on the expression of heat shock protein (HSP), redox regulation and endoplasmic reticulum (ER) stress markers in tibialis anterior (T.A.), and soleus muscle of mice. Aging was associated with a lower thioredoxin-1 (TRX-1) to thioredoxin-interacting protein (TxNiP) ratio, a determinant of redox regulation, and an increased indicator of ER stress, related to apoptosis signaling in both muscles. Long-term exercise decreased TxNiP in T.A. and soleus muscles and increased the TRX-1/TxNiP ratio in soleus muscle of aged mice. Inducible HSP70 and constituent HSC70 were upregulated, whereas ER stress was reduced after exercise in soleus muscle. Their data demonstrated that aging induces oxidative stress and activates ER stress-related apoptosis signaling in skeletal muscle, whereas long-term wheel running improves redox regulation, ER stress adaptation, and attenuates ER stress-related apoptosis signaling, suggesting that life-long exercise can protect against age-related cellular stress.

Zhao et al. evaluated the rehabilitative effects of swim training following stable myocardial infarction (MI) in aged mice. They compared the effects of swimming training (ST) of different durations (15 and 60 mins once per day for five days a week for 8 weeks) on cardiac function and mitochondrial quality and examined a potential role for Sirtuin (SIRT) 3 for the rehabilitative process. The main finding of such paper is that 15 mins of ST, rather than 60 min of ST, significantly augmented left ventricular function, increased survival rate, and suppressed myocardial fibrosis and apoptosis. Moreover, it improves mitochondrial morphology regulating mitochondrial fission-fusion signaling and regulated mitophagy signaling via inhibiting LC3II and P62 levels and increasing PINK/Parkin expression. Moreover, 15 mins ST inhibited ROS production and enhanced antioxidant SOD2 activity. Notably, 15 mins ST significantly increased Sirtuin (SIRT) 3 level *in vivo*. It is likely that the positive regulation induced by short-duration ST regimen on elevated SIRT3 protein level, improved mitochondrial quality control, and decreased apoptosis and fibrosis contributing to the observed more resistant phenotype.

In the whole, the papers reported in this special issue furnish new inside on the protective role of physical activity for the maintaining of health during aging and illness.

*Paola Venditti* *Yong Zhang* *Zsolt Radák* *José Magalhães*

